# Cell Therapy: A Safe and Efficacious Therapeutic Treatment for Alzheimer’s Disease in APP+PS1 Mice

**DOI:** 10.1371/journal.pone.0049468

**Published:** 2012-12-03

**Authors:** Neel R. Nabar, Fang Yuan, Xiaoyang Lin, Li Wang, Ge Bai, Jonathan Mayl, Yaqiong Li, Shu-Feng Zhou, Jinhuan Wang, Jianfeng Cai, Chuanhai Cao

**Affiliations:** 1 Department of Pharmaceutical Sciences, College of Pharmacy, University of South Florida, Tampa, Florida, United States of America; 2 USF-Health Byrd Alzheimer’s Institute, University of South Florida, Tampa, Florida, United States of America; 3 Chinese People Liberty Army General Hospital, Beijing, China; 4 Third Military Medical University, Chongqing, China; 5 Department of Chemistry, University of South Florida, Tampa, Florida, United States of America; 6 Tianjin Huanhu Hospital, Tianjin, China; University of North Dakota, United States of America

## Abstract

Previously, our lab was the first to report the use of antigen-sensitized dendritic cells as a vaccine against Alzheimer’s disease (AD). In preparation of this vaccine, we sensitized the isolated dendritic cells *ex vivo* with Aβ peptide, and administered these sensitized dendritic cells as a therapeutic agent. This form of cell therapy has had success in preventing and/or slowing the rate of cognitive decline when administered prior to the appearance of Aβ plaques in PDAPP mice, but has not been tested in 2×Tg models. Herein, we test the efficacy and safety of this vaccine in halting and reversing Alzheimer’s pathology in 9-month-old APP+PS1 mice. The results showed that administration of this vaccine elicits a long-lasting antibody titer, which correlated well with a reduction of Aβ burden upon histological analysis. Cognitive function in transgenic responders to the vaccine was rescued to levels similar to those found in non-transgenic mice, indicating that the vaccine is capable of providing therapeutic benefit in APP+PS1 mice when administered after the onset of AD pathology. The vaccine also shows indications of circumventing past safety problems observed in AD immunotherapy, as Th1 pro-inflammatory cytokines were not elevated after long-term vaccine administration. Moreover, microhemorrhaging and T-cell infiltration into the brain are not observed in any of the treated subjects. All in all, this vaccine has many advantages over contemporary vaccines against Alzheimer’s disease, and may lead to a viable treatment for the disease in the future.

## Introduction

Already, one out of every eight older American suffers from Alzheimer’s Disease (AD), a devastating age-related neurodegenerative disease that manifests itself clinically as a steep decline in cognitive function [1]. With vast improvements in healthcare now available to the general population, the incidence of AD is expected to increase as average life expectancy rises. In fact, it has been estimated that the prevalence of AD worldwide will quadruple by 2050, at which point more than 100 million people will be affected [2]. Though a great deal of research has focused on the development of AD therapeutics, there are still no disease-modifying drugs on the market [3], [4].

The long since proposed amyloid cascade hypothesis of AD has implicated the amyloid-beta (Aβ) peptides in pathogenesis of the disease, as aggregates of Aβ_1–40_ and Aβ_1–42_ are the major constituents of the hallmark insoluble plaques found in brains of AD patients upon histological analysis [5]. The aggregation of these peptides, along with hyperphosphorylation of the tau protein, are now generally accepted as the major causative factors of the neuronal loss associated with Alzheimer’s progression [6]–[8]. As such, many have considered Aβ immunotherapy as a therapeutic approach for the treatment of this disease. Around the turn of the millennium, three independent reports of an active vaccine consisting of an Aβ peptide and adjuvant were published in *Nature* [9]–[11]. These reports all indicated that active vaccination effectively elicited an antibody titer and improved cognitive function in Alzheimer’s mice, likely through successful plaque removal in the brains of these mice. Moreover, passive immunotherapy in murine models has shown similar results with regards to treatment efficacy [12], [13], indicating that the presence of anti-Aβ antibodies works to both effectively inhibit Aβ deposition and clear Aβ plaques in the brain.

Unfortunately, the phase II clinical trials (Elan’s AN-1792) with an active vaccine (peptide plus adjuvant) had mixed results. The study was suspended prior to the completion due to 6% of patients developing meningoencephalitis after multiple vaccinations, though the vaccine did show some clinical benefit [14]–[16]. Follow up studies have associated the observed brain inflammation with initiation of a Th1 inflammatory cascade, due in part to the presence of an adjuvant. Since then, scientists in both academia and industry have investigated methods to circumvent production of the Th1 response, which has lead to four active anti-Aβ immunotherapies currently in clinical trials [17]–[19]. Earlier, our lab reported that Aβ peptides have both major immunogenic B-cell and T-cell epitopes [20]. Vaccines currently in clinical trials use only the B-cell epitope of Aβ in order to avoid the T-cell induced Th1 inflammatory response seen in AN-1792 [21], [22].

Our lab has focused on development of an anti-Aβ vaccine that makes use of *both* the T-cell and B-cell epitope, while avoiding the Th1 related inflammation seen in AN-1792. Specifically, we have considered administration of antigen sensitized dendritic cells (DCs) as a therapeutic approach. Antigen sensitized DCs have resulted in vaccines with progression into clinical trials for many diseases, and have circumvented vaccine related inflammation in the past [23]–[27]. Moreover, DCs cultured with IL-4 promote a Th2 anti-inflammatory response and inhibit the Th1 inflammatory response [28] - advantageous characteristics for AD vaccine development. In the past, our lab has shown that dendritic cells sensitized with an Aβ peptide that has two point mutations in the T-cell epitope of Aβ are able to elicit a notable antibody titer in BALB/c, APP_sw_, and PDAPP mice. Moreover, we have shown that this antibody titer results in significant reduction of Aβ load and corresponding behavioral improvements [29], [30].

In this study, we aim to investigate the immunogenic effects of a variety of mutated Aβ peptides on DCs in vitro. We then look to use the peptide that generates an optimal immune response when sensitized dendritic cells for use in our vaccine, and characterize the vaccine in APP+PS1 2×transgenic (Tg) Alzheimer’s mice with regards to its, (1) ability to elicit an antibody titer, (2) ability to avoid the Th1 inflammation seen in AN-1792, and (3) efficacy in improving cognitive function. Finally, we hope to gain some insight into the mechanisms behind AD progression by investigating the immunogenic effects of this vaccine.

## Materials and Methods

### In Silico Prediction of Aβ Peptide Binding Affinity to HLA a-0201

The HLA peptide binding affinity tool (available at http://www-bimas.cit.nih.gov/molbio/hla_bind/) was used to determine the affinity of different Aβ regions to the HLA a-0201 molecule, the most common human HLA [31]. After determination of the probable T-cell binding region, this tool was used to evaluate the binding affinity of Aβ molecules with point mutations in the T-cell epitope. Peptide sequences are listed in Table 1.

**Table 1:** Sequences of Aβ1–42 peptide with mutated T cell epitope.

Peptide Name	Sequence
Aβ 1–42 (PWT)	DAEFRHDSGYEVHHQKLVFFAEDVGSNKGAIIGLMVGGVVIA
Aβ 1–42 (PFM) with Flemish mutation	DAEFRHDSGYEVHHQKLVFF**G**EDVGSNKGAIIGLMVGGVVIA
Aβ 1–42 (PDM) with Dutch mutation	DAEFRHDSGYEVHHQKLVFFA**Q**DVGSNKGAIIGLMVGGVVIA
Aβ 1–42 (PDFM) with Flemish and Dutch Mutations	DAEFRHDSGYEVHHQKLVFF**GQ**DVGSNKGAIIGLMVGGVVIA
Aβ 1–42 (P22W) with mutation at 22	DAEFRHDSGYEVHHQKLVFFA**W**DVGSNKGAIIGLMVGGVVIA
Aβ 1–42 (P24M) with mutation at 24	DAEFRHDSGYEVHHQKLVFFAED**G**GSNKGAIIGLMVGGVVIA

### Immunogenic Effect of Aβ Peptides *in vitro*

#### Bone marrow isolation

Bone Marrow Isolation followed an established protocol, published in both Cao et al. and Luo et al [29], [30]. In brief, bone marrow was removed from 7–11 week old female C57BL/6 mice and the femurs cleanly excised, all excess tissues were removed. Shortly thereafter, the bones were merged in cold phosphate-buffered saline (PBS), washed with ethanol, and again soaked in 1×PBS. After the ends of each femur were cut, bone marrow was flushed with medium (99% RPMI and 1% Antibiotics). Bone marrow was then gently re-suspended and passed through 70 µm sieves into a centrifuge tube. The mixture was centrifuged for 10 min at 1100 rpm, followed by removal of the supernatant and brief vortexing of the pellet. 5 ml ACK (0.15 M NH_4_Cl, 1 mM KHCO_3_, 0.1 mM EDTA, pH 7.3 at room temperature) was used for Red blood cells lysis with an incubation period of 30 sec while shaking, and lysis was stopped by the addition of 45 ml HBSS. After centrifugation at 1,100 rpm (10°C for 10 min), cells were suspended at 1×10^6^ cells/ml in medium(RPMI with+10%-FBSand 1% Antibiotics). Cells were cultured in 6 well plates.

#### Peptide preparation

Wild Type Aβ1–42 (PWT), Aβ with the Dutch mutation (PDM), Aβ with the Flemish Mutation (PFM), Aβ with the Dutch and Flemish mutations (PDFM), Aβ with a tryptophan substituted for a glutamic acid residue at position 22 (P22W), and Aβ with a glycine substituted for a valine residue at position 24 (P24M) were synthesized by Biomer Tech (CA, USA) and used to sensitize DCs as follows.

#### Sensitization of peptide to DCs

On the second day of DC culturing, the medium was completely aspirated to remove all non-adherent cells (lymphocytes, progenitors, etc.), and 3 ml of fresh DC culture medium was added. The cells were allowed to grow in a CO_2_ incubator (5% CO_2_) and on the day 4 were treated as follows: 1 ml/well old medium was aspirated and replaced by 1 ml/well fresh DC culture medium containing 60 µg/ml peptide (diluted to a final concentration 20 µg/ml). On the 8^th^ day, DCs were harvested, and the supernatant collected for analysis.

#### Cytokine expression detection

The immunogenic profile of each peptide was investigated with determination of a complete cytokine expression profile using a Luminex Liquidchip system (Panomics, CA, USA) following each step of the Liquidchip protocol as previously described [29]. Levels of IL-1α, IL-1β, IL-2, IL-3, IL-4, IL-5, IL-6, IL-9, IL-10, IL-12, IL-13, IL-15, IL-16, G-CSF, GM-CSF, IFN-γ, TNF-α, RANTES, Eotaxin, KC, MCP-1, MIP-1α, and MIP-1β were measured in the supernatant.

### *In vivo* Animal Testing

#### Ethics statement

This study was carried out in strict accordance with the recommendation in the Guide for the Care and Use of Laboratory Animals of the National Institutes of Health. The protocol was approved by the Institutional Animal Care and Use Committee (IUCAC) of the University of South Florida (Permit Number: R3337).

#### Grouping

After PDFM was selected as the optimal peptide for use in our DC vaccine, 9.5-month old Tg-APP+PS1 mice (n = 18) mice and aged matched C57BL/6 non-Tg mice (n = 6) were obtained from the USF Health Byrd Alzheimer Institute. The APP+PS1 mice were produced from a cross between mutant APP_K670N,M671L_ Tg line Tg 2576 and mutant PS1 Tg line and were of mixed background (C57 = 28%, B6 = 58%, SW = 6%, SJL = 8%) [32]. After determination of the optimal peptide for DC vaccine preparation, the Tg mice were randomly divided into 3 groups: the Tg PDFM, Tg-PWT, and the Tg Control groups. Non-Tg mice made up the PDFM group. Each group contained 6 mice. Mice of the PDFM groups were vaccinated with PDFM sensitized dendritic cells, while mice of the Control group received PBS injections. The PDFM groups were active vaccine groups, while the PBS group was the negative control and should not elicit an antibody titer.

### Vaccine Preparation

#### DC preparation

DC preparation followed the same protocol as described above. However, after centrifugation and suspension at 1×10^6^ cells/ml in RPMI medium (with10% FBS and 1% Antibiotics), 10 ng/ml GM-CSF and 10 ng/ml IL-4 (BD-Pharmgen, San Jose CA) were added to the media, and cells were cultured in 6-well plates (3 ml/well).

#### Sensitization of peptides to DCs

Wild Type Aβ1–42 (PWT) and Aβ with the Dutch and Flemish mutations (PDFM) were synthesized by Biomer Tech (CA, USA) and used to sensitize DCs from day 1 to day 7 as previously described. On the 8^th^ day, DCs were harvested, washed three times with 1×PBS, and the concentration adjusted to 5.0×10^6^ cells/ml.

### Vaccine Administration Schedule

Mice of the PDFM groups were administered 1.0×10^6^ cells in 200 µl 1×PBS via I.P. injection during each treatment. The DC percentage of injected cells was more than 90%. Mice of the PBS group were given 200 µl PBS via IP injection at the same time points. Mice were injected a total of 6 times. The first injection was given on day 1, and booster injections were given using 20-day intervals starting on the day 30. Injections were administered on Days 0, 30, 50, 70, and 90.

### Sample Collection Schedule

Blood samples were collected in EDTA tubes by submandibular phlebotomy bleeding. For each sample, plasma was isolated by centrifugation at 1,000×*g* for 3 min, transferred into screw-capped tubes, and stored at −80°C. Mice were bled pre-immunization and 7 days after each injection (except for the first injection, blood was drawn 10 days after the first injection). An additional blood draw was taken on day 24 in order to characterize longevity of the antibody titer response following the first injection. In total, animals were bled on Days 0, 10, 24, 37, 57, 77, and 97.

### Aβ Antibody Titer Determination

An ELISA method was used to determine antibody levels in plasma samples using Aβ1–42 peptide as the coating antigen. After serum isolation via centrifugation, 96-well plates were coated with 50 µl/well Aβ 1–42 in CBC buffer at 10 µg/ml and a CBC plate was set up to determine background binding. Then, both Aβ and CBC plates were incubated at 4**°**C overnight. After 5 washes with a wash buffer, plates were subject to a blocking step with 180 µl blocking buffer (1×PBS containing 1.5% BSA) for 45 min, then washed an additional 5 times with wash buffer. Samples diluted with blocking buffer were added to both Aβ plates and CBC-plates, with two-fold serial dilutions starting at 1∶200, and incubated at 37**°**C for 1 hr., followed by 12 washes with wash buffer. HRP-conjugated anti-mouse IgG was loaded into each well at 1∶5000 dilution with dilution buffer, incubated for 1 hr at 37**°**C, then washed 12 times. TMB substrate was dissolved in PCB buffer and 100 µl was added into each well. The colorimetric reaction was stopped with 25 µl 2 N H_2_SO_4_. Plates were read at 450 nm/620 nm with a BioTek Synergy Reader. Samples having readings three times higher than controls were considered positive; the highest dilution was considered the endpoint titer.

### Inflammatory Response Characterization

#### Cytokine expression detection

Cytokine expression was detected using a Luminex Liquidchip system (Panomics, CA) following each step of the Liquidchip protocol as previously described [29]. Levels of IL-4, IL-10, G-CSF, IFN-γ, and TNF-α were measured in the serum.

#### Ig isotyping

Ig isotyping was determined using the Invitrogen Mouse Immunoglobulin Isotyping Kit by Life Technologies (Frederick, MD) and following manufacturer’s instructions. Ig isotyping allows calculation of an IgG1/IgG2a ratio, which helps to differentiate Th1 from Th2 responses in vaccinated mice. As IgG1 is driven by IL-4 (Th2 cytokine), and IgG2a is driven by IFN-γ (Th1 cytokine) [33], an increase in post-vaccination ratio indicates a Th2 response, and a decrease in post-vaccination ratio indicates a Th1 response. Both total and Aβ -specific Ig isotyping was done in this study, though only total Ig isotyping can be done pre-vaccination.

### Behavioral Testing – radial Arm Water Maze

#### Apparatus

The Radial Arm Water Maze (RAWM) system was a small pool (diameter: 90 cm, depth: 50 cm) with an aluminum insert to create 6 radially distributed swim arms. An escape platform 10 cm in diameter and 1.5 cm below the surface of the water can be located in any of these arms. The water was kept between 25–27°C and made opaque, enabling the platform to be hidden from sight. The maze apparatus was placed in a room with dim lighting, and many extra maze cues were present on the walls and surroundings that rats could use for navigation of the maze.

#### Procedure

Mice were tested on day 105 after the initial vaccine treatment using the RAWM; the mice were nearly 13 months old. One day before behavioral testing, all mice were allowed a swimming acclimatization session to reduce stress during behavioral testing. All mice were handled gently and with care to avoid unnecessary stress on the mice. Starting on the day 105, each mouse was given 5 one-minute trials daily for 12 days; a 30-minute delayed retention trial (T5) was used as indices of working memory. On any given day of testing, the submerged clear platform was placed at the end of one of the six swim arms. The platform location was changed daily to a different arm in a random pattern. On each day, different start arms for each of the 5 daily trials was randomly selected, with the stipulation that the start arm cannot be the same as the arm the platform is in. For any given trial, the mouse was gently placed into that trial’s start arm and given 60 seconds to find the platform. Each time the mouse entered a non-platform containing arm, it was gently pulled back into the start arm and an error recorded. Both the number of errors (incorrect arm choices) and escape latency were recorded for each daily trial. After the T4 acquisition trial, the mouse was dried with a towel and returned to its cage for 30 minutes prior to the T5 retention test. Performance on each trial was videotaped and analyzed with image tracking software (HVS Image, Hampton, UK), which provided dependent measures such as latency (s) and speed (cm/s). Also, the order of entry into the maze arms was recorded so that the number of errors could be analyzed. The apparatus and procedure is described in detail in Arendash et al [34].

### Histopathological Analysis

#### Brain sample collection and preparation

After behavioral testing, the mice were anesthetized with chloral hydrate (0.01 ml/g by i.p. injection) and perfused through the left ventricle with saline until the liver changed color from red to white. The brains were then removed and immersion fixed in fresh periodate-lysine-paraformaldehyde (PLP). After fixation, the brains were imbedded in paraffin and stored until sectioning.

#### Brain Aβ staining and analysis

Congo Red staining was performed on 4 µm frozen sections from the hippocampus (CA1, CA3, and DG) and cortex (CX and ECX) using the procedure described in Morgan et al [10]. The area occupied by the stain was measured with a Videometric V150 image analysis system (Oncor) on a Nikon Microphoto FX microscope. An unbiased experimenter (one unaware of the subject condition) measured the stain regions using HIS segmentation. The experimenter measured both stain intensity and area, though only stain area is reported here as is the convention for measurement of insoluble Aβ burden.

#### CD3 staining

4 µm frozen sections from the hippocampus (CA1, CA3, and DG) and cortex (CX and ECX) were stained with mouse anti-CD3antibody after a mouse IgG blocking step. The immunostaining procedure is previously described in Postupna et al [35].

#### Prussian blue staining

4 µm frozen sections from the hippocampus (CA1, CA3, and DG) and cortex (CX and ECX) were stained with Prussian Blue to examine brain vasculature for bleeding.

### Plasma Aβ Level

Plasma Aβ-levels were tested pre-immunization and on the day 77 using a Luminex assay (Biosource Cat#LNB0001) according to the manufacturer’s protocol. Briefly, Luminex capture beads were prepared and 50 µl detection antibody was added to each well. 25 µl sample and 25 µl peptide assay diluent were also added to each well, and 50 µl standard was added to the designated standard wells. The mixture was allowed to incubate for 3 hrs at RT on an orbital shaker. After the incubation period, the plate was thoroughly washed and 100 µl 1×RPE added to each well. The samples were read using Luminex after an additional 30-min incubation period.

### Statistical Analysis

All statistical analysis was performed using Graphpad Prism (Prism 5.01 for Windows) and data are presented as Mean±SEM. Analysis of antibody titers and cytokine expression between groups was done using a Two-Way ANOVA followed by Bonferroni post-tests. Analysis of Aβ specific Ig-Isotyping and Congo Red staining were done using ANOVA with ad hoc Bonferroni post-tests. A two-tailed Student’s T-Test was use for analysis of plasma Aβ and total Ig Isotyping. P-values less than 0.05 were considered statistically significant.

## Results

### Peptide Selection

#### T-Cell epitope binding prediction

The *in silico* binding affinity of Aβ was calculated using 9 different amino acids as start points. The binding affinity for Aβ was highest when amino acid 16 (a lysine residue) was used as the start point, suggesting that this region serves as the *in vivo* T-cell epitope (Table 2). After determination of the T-cell epitope, the binding affinities of different mutations on the T-cell epitope were calculated. P22W had the highest HLA a-0201 binding score, while PDM and PDFM had medium binding scores. The wild-type and PFM peptides had less affinity than PDFM and PDM, but both had a significantly higher affinity than P24M. Peptides with a high binding score (P22W), medium binding score (PDM, PDFM, PFM, and wildtype), and low binding score (P24M) were used for the *in vitro* study. Peptides with medium binding scores are optimal for vaccine development. These peptides are expected to elicit an antibody titer through successful antigen presentation, while avoiding an overly robust T-cell response resulting in inflammation. The results are shown in Table 3.

**Table 2:** Calculated T-cell binding affinities for different Aβ start sites.

Start position on Aβ peptide	AA sequences	HLA/peptide binding
AA16	KLVFFAEDV	453.27
AA33	GLMVGGVVI	15.827
AA10	YEVHHQKLV	6.221
AA34	LMVGGVVIA	5.752
AA31	IIGLMVGGV	4.861
AA28	KGAIIGLMV	1.589
AA4	FRHDSGYEV	0.182
AA32	IGLMVGGVV	0.152
AA24	VGSNKGAII	0.047

**Table 3:** Calculated binding affinity for mutated Aβ peptides.

Name of mutation	Binding score	Amino acid sequence
Wild type	453.27	KLVFFAEDV
Flemish mutation	453.27	KLVFF**G**EDV
Dutch Mutation	925.042	KLVFFA**Q**DV
Flemish and Dutch	925.042	KLVFF**GQ**DV
p22W	6937.812	KLVFFA**W**DV
p24M	0.486	KLVFFAED**G**
p22F	5365.241	KLVFFA**F**DV
P22R	185.008	KLVFFA**R**DV
P24E	0.097	KLVFFAED**E**

#### PWT presentation in DCs modulates cytokine expression

**Figure 1:**
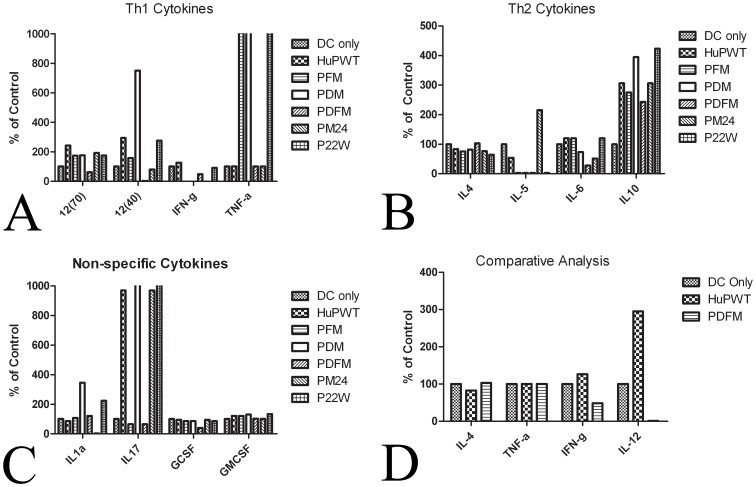
Cytokine response post *in vitro* antigenic stimulation. Wild type and mutated Aβ peptides were sensitized to DCs for 5 days: A) Th1 Cytokines B) Th2 Cytokines C) Non-Specific Cytokines D) Comparative Analysis. PDFM was the only peptide to downregulate TNF-α and IFN-γ, while upregulating IL-4.

Dendritic cells cultured with wild-type Aβ peptide exhibited differential cytokine expression in comparison with the DC only control. Our study showed PWT presence increased IL-12 (70), IL-12 (40), and IFN-γ production, while decreasing IL-4 production. Interestingly, PWT did not cause an immediate increase in TNF-α, though other Th1 cytokines were increased (Figure 1).

#### PDFM elicits the optimal cytokine response for use in a DC vaccine

Prior AD vaccines have faced problems due to generation of a Th1 response, thus we selected a peptide which upregulated Th2 cytokine production and downregulated Th1 cytokine production during presentation to DCs. As IFN-γ and TNF-α drive Th1 T-cell differentiation, and IL-4 drives Th2 specific differentiation, we selected PDFM for use in our DC vaccine as its cytokine expression profile was indicative of the immune response our vaccine should generate. All synthetic peptides (besides PDFM and P24M) increased levels of TNF-α. However, compared to PDFM and P24M, PDFM showed a marked increase in IL-4, while P24M actually decreased IL-4 production. Although, both PDFM and P24M decreased IFN-γ production in comparison to the control, only the sensitization with PDFM inhibited IL-12(70) and IL-12(40). Expression levels of select cytokines are shown in Figure 1.

### Vaccine Efficacy

#### DC Vaccine induces lasting antibody response in both Tg and Non-Tg mice

**Figure 2:**
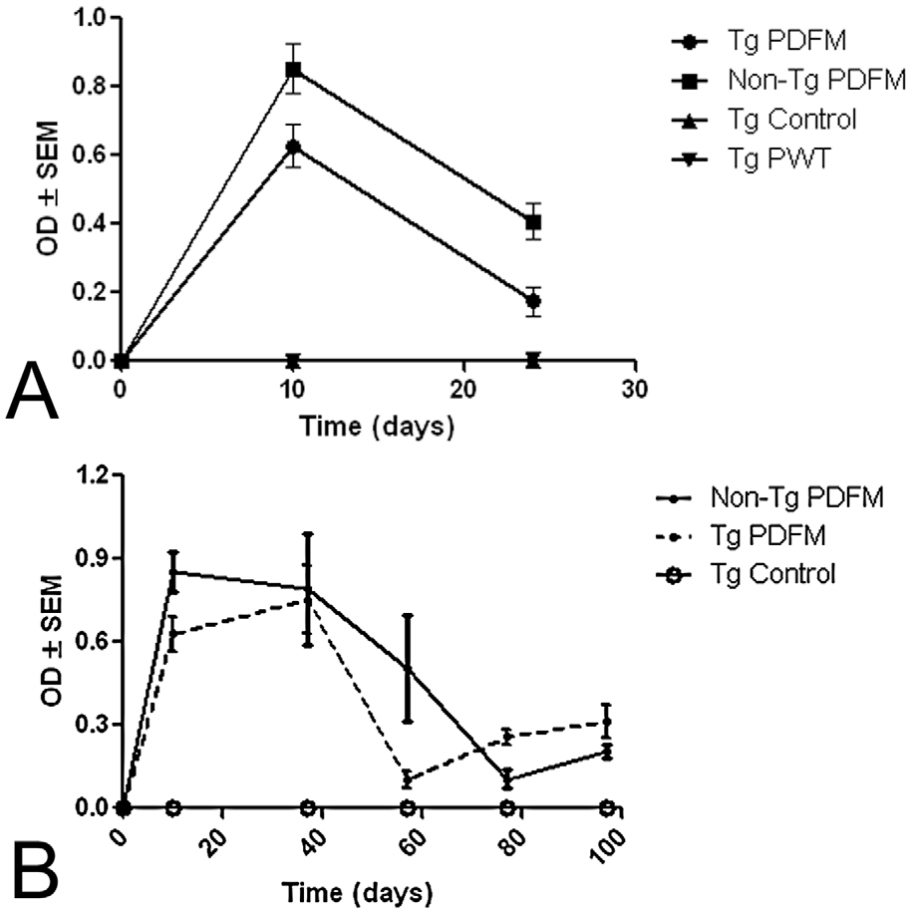
Antibody titer post vaccination. A) short term response. Mouse of the Tg PDFM and Non-Tg PDFM groups elicited a significant antibody titer both 10 and 24 days post vaccination, but the Tg control and Tg PWT group did not (*P*<0.001). The Non-Tg PDFM group was significantly higher than the Tg PDFM group at both time points (*P*<0.001). B) Long term antibody response. Both groups had positive antibody titers throughout the duration of the experiment. Antibody titers for both groups showed a significantly elevated titer compared to the control at all time points except day 77 (*P*<0.05). All readings were captured at 450 nm with 1∶1000 diluted samples.

10 days after the initial vaccination, antibody titers of the Non-Tg PDFM and Tg PDFM were significantly higher than both the control and pre-vaccination titers (ANOVA, Bonferroni post-tests, *P*<0.001). Mice of the Tg PWT group did not show an antibody response, and did not have a significantly different titer from the control (Figure 2A). As such, these mice were removed from the study. Sensitized DCs, even when sensitization occurs *ex vivo*, are able to process and present antigens to the appropriate effectors cells via traditional antigen presentation pathways. However, when a DC captures a self-antigen, antibody production is suppressed – explaining the absence of an antibody titer when mice were treated with PWT sensitized DCs. The PDFM group was able to elicit an antibody titer because the PDFM peptide contains 2 mutations in the T-Cell Epitope, and therefore is not recognized as a self-antigen.

In order to determine the longevity of the elicited antibody titer, antibody titers were measured again at the day 24 after initial vaccination. The titers of both groups dropped when compared to Day 10 (*P*<0. 001), but the Non-Tg PDFM (*P*<0.001) and Tg PDFM (*P*<0.01) still had a significantly elevated antibody titer compared the control. Moreover, the Non-Tg PDFM group appeared to have a more robust antibody response, as antibody titers in this group were significantly higher than that of the Tg PDFM group at both time points (*P*<0. 001).

As antibody titer lowered significantly between Day 10 and 24, booster injections were given every 20 days starting on Day 30. Mice of the Tg PDFM group were positive for an antibody titer throughout the duration of the experiment, with a mean reading of.279 OD. The antibody titer peaked at Day 10, and decreased slightly by Day 37, though still significantly higher than the control (ANOVA, Bonferroni post-tests, *P*<.001). The antibody titer decreased again on Days 57 and 77, and once again increased to levels significantly higher than the control by Day 97 (*P*<.05).

The Non-Tg PDFM group also showed an antibody titer throughout the duration of the experiment, with a mean reading of 0.373 OD. The antibody titer peaked at Day 10 and remained significantly higher than the control until day 57 (*P*<0.001). At day 77, the antibody titer decreased, but increased again at Day 97, at which point it was significantly higher than the control (*P*<0.05). There were no significant differences between mice of the Tg PDFM and Non-Tg PDFM groups except on Days 10 and 57 - mice of the Non-Tg PDFM group had significantly higher antibody titers (*P*<0.001). Mice of the Tg Control group did not show an antibody titer throughout the duration of the experiment (*P*<0.001) (Figure 2B).

#### Mice 8 and Mice 14 of the Tg PDFM group were low responders

**Figure 3:**
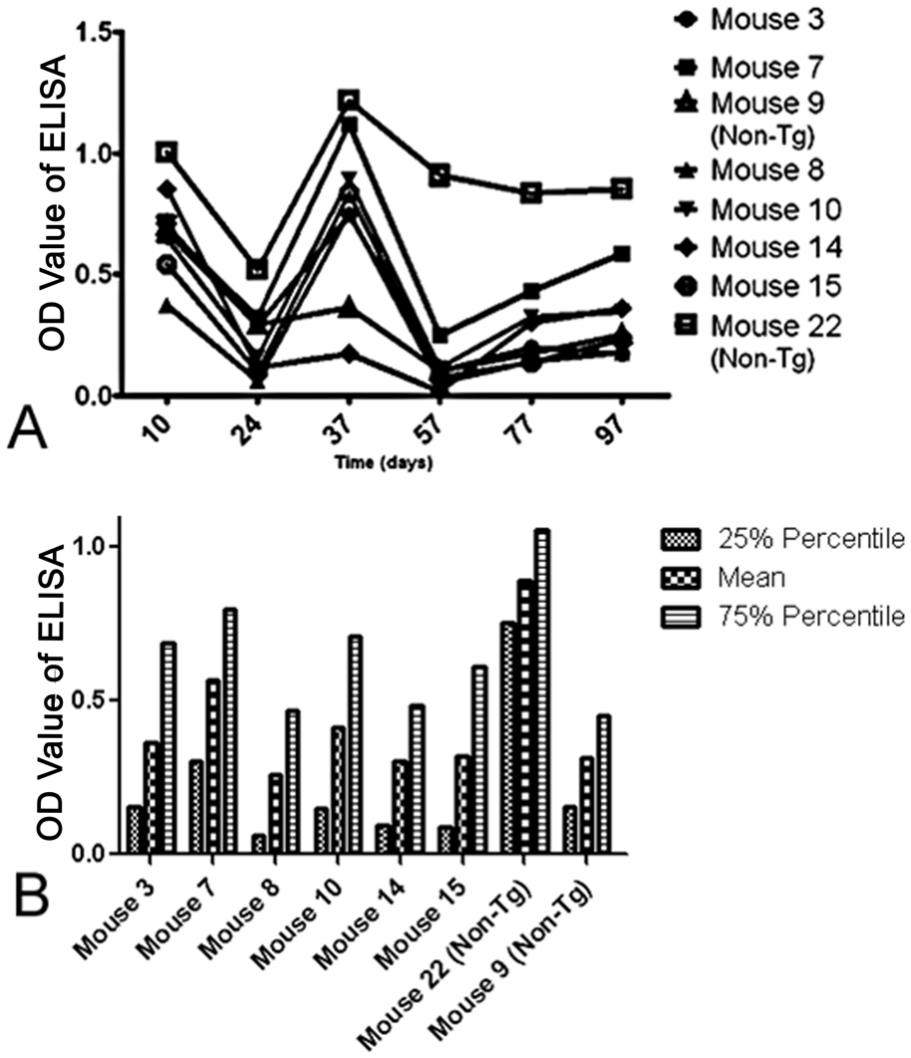
Antibody response of individual mice. A) Antibody responses on all Tg and select Non-Tg mice are shown. B) The mean and 75^th^ percentile value was used to determine level of antibody response. Mice 8 and 14 did not have a robust, sustained antibody response and were deemed low responders.

As shown by Vellas et al. in a long-term follow up study to AN-1792, antibody response varied greatly between individuals. Responders to the vaccine showed a significant increase in cognitive function, while non-responders did not [15]. In analyzing the antibody titers of the individual mice (Figure 3A), we used the mean and 75^th^ percentile antibody titer value as an indicator of the strength and longevity of the antibody response. Any mouse with a mean antibody titer less than one standard deviation away from 0, and a 75^th^ percentile value below.5 OD, was considered a low responder (Figure 3B). Mice 8 and 14 met the low responder criterion.

#### PDFM vaccine decreased brain Aβ plaque levels

**Figure 4:**
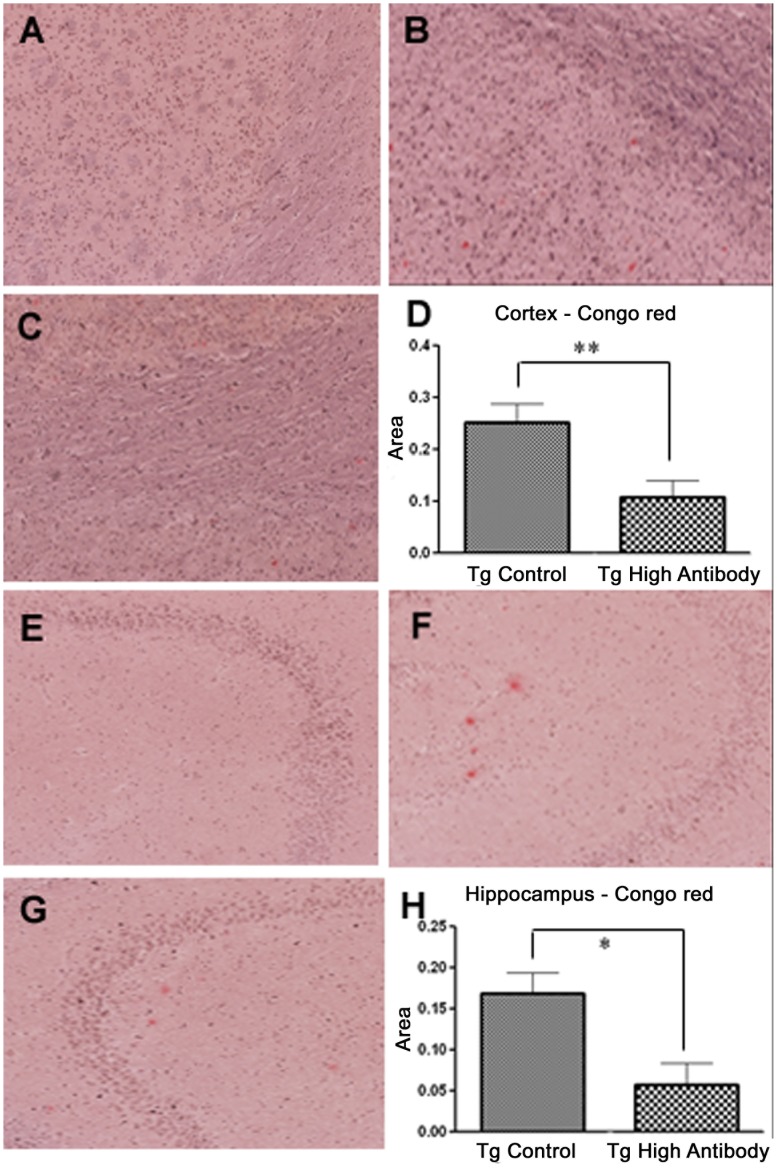
Congo red staining. A) Non-Tg PDFM Cortex B) Tg Control Cortex C) Tg PDFM Cortex D) Cortex Aβ Burden E) Non-Tg PDFM Hippocampus F) Tg Control Hippocampus G) Tg PDFM Hippocampus H) Hippocampal Aβ Burden. Aβ Burden was significantly decreased in the Tg PDFM group compared to the control group in the Cortex (57.5%) and the Hippocampus (65.9%) (*P<*0.05). No plaques were observed in Non-Tg mice. All images were captured at 10×magnification.

Brain Aβ burden was determined via Congo red staining of the cortex and hypothalamus. Overall, Aβ burden between groups appeared to be significantly different (ANOVA, *P* = 0.0105). Mice with High Antibody titer in the Tg PDFM group showed less Aβ accumulation in the cortex (57.5% decrease, ANOVA, Turkey’s Multiple Comparison Test, *P*<0.01) and in the hippocampus (65.9% decrease, *P*<0.05) compared to the Tg Control Mice. Mice with High Antibody Titer in the Tg PDFM group also showed 42.2% and 34.3% decreases in Aβ accumulation in the cortex and hippocampus respectively compared to mice of the Low Antibody Tg PDFM, though neither difference reached statistical significance. No amyloid plaques were observed in Non-Tg mice. Congo red staining results are shown in Figure 4.

#### PDFM vaccine modulates blood Aβ levels

**Figure 5:**
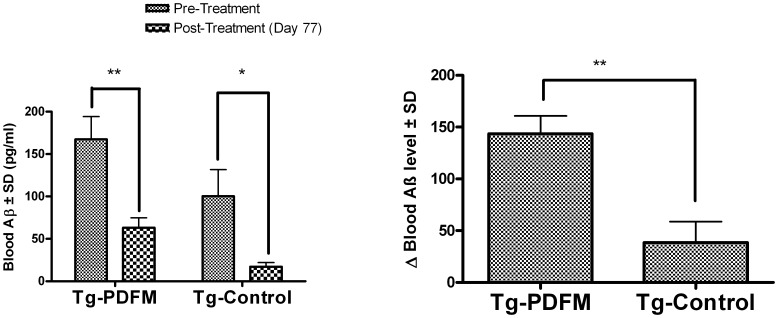
Blood Aβ. A) Pre-Treatment (Day 0) vs. Post-Treatment (Day 77). Blood Aβ levels decreased significantly from Day 0 to 77 in both Tg Groups (*P*<0.05). B) Change in Blood Aβ. Mice of the Tg PDFM group had a greater decrease in Blood Aβ than those of the control group (*P*<0.01).

As demonstrated by Trinchese et al., blood Aβ levels decrease slightly in APP+PS1 mice with age [36]. Our results are consistent with this study, in that mice of the Tg Control group had a small but significant (*P*<0.05) drop in blood Aβ levels from Day 0 to Day 77. Mice of the Tg PDFM group also had a drop in blood Aβ levels (*P*<0. 01), though the change was significantly more in the Tg PDFM group than in the Tg Control group (*P*<0.01) – as shown in Figure 5.

#### PDFM treatment may provide cognitive benefit

**Figure 6:**
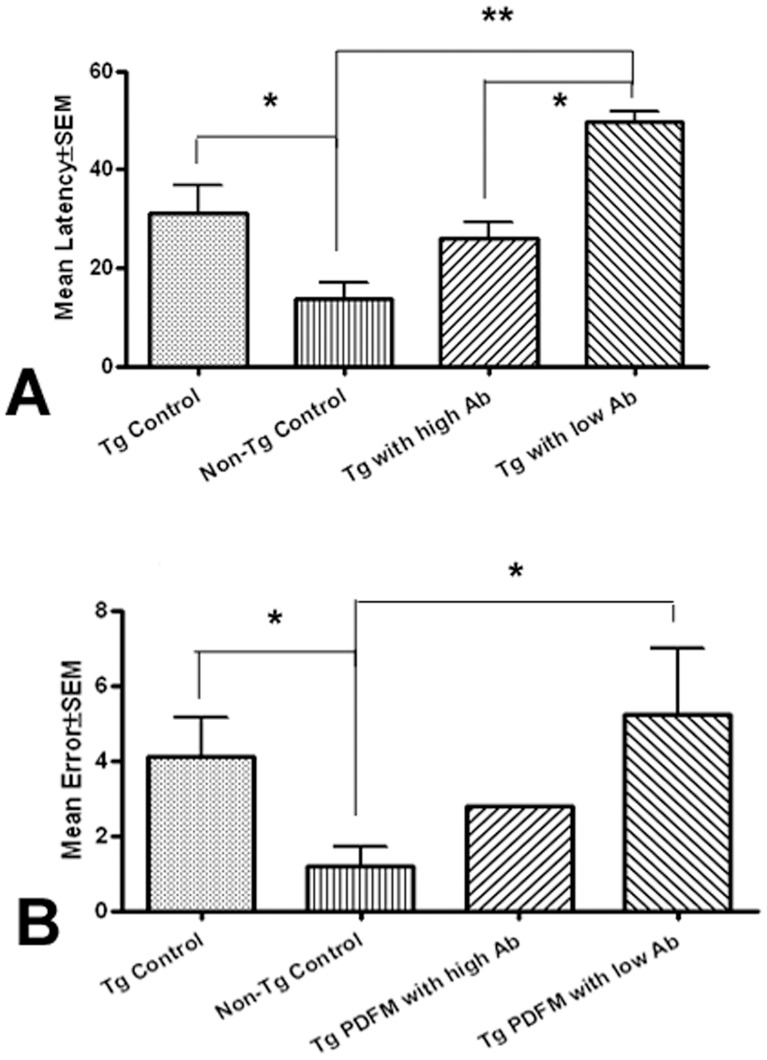
Behavioral results. A) Latency. The Tg PDFM with high antibody titer group performed moderately better than the Tg control group, though not reaching statistical significance. The low-responders performed significantly worse than the Tg Control (*P*<0.05). B) Error. The Tg PDFM with High Antibody Titer group performed slightly better than the Tg Control Group, though not significant. The low-responders performed significantly worse than the Tg control (*P*<0.05).

Cognitive function was measured using the T5 trails of the RAWM as an index of working memory. The composite results from all blocks are shown in Figure 6. As expected, Non-Tg mice performed significantly better than the Tg Control with regards to both latency and errors (*P*<0.05). With regards to latency, mice of the Tg PDFM group with High Antibody Titer performed moderately better than Tg Control mice, though the difference did reach statistical significance (*P* = .19). Interestingly, mice of the Tg PDFM Group with Low Antibody Titer performed significantly worse than the Tg Control mice (*P*<0.05) (Figure 6A). As shown in Figure 6B, mice of the treatment group (Tg PDFM with High Antibody Titer) had fewer errors in maze navigation than mice of the Tg Control group, though again the difference did not reach statistical significance (*P* = 0.15). Once again, mice of the Tg PDFM group with Low Antibody Titers performed significantly worse than Tg Control Mice and Tg PDFM mice with High Antibody Titers (*P*<0.05).

### Vaccine Safety Evaluation

#### PDFM does not elicit a Th1 inflammatory response

Prior to treatment (Day 0), levels of all cytokines measured were not significantly different between groups. In accessing differences between cytokine expression profiles after a long-term treatment, serum samples from Day 77 were assayed. In Tg mice, levels of IL-4, IL-10, TNF-α, and IFN-γ were significantly increased after the PDFM treatment (Two-Way ANOVA, Bonferroni’s Posttests, *P*<0.001). Mice of the Non-Tg PDFM group had significant increase IL-4, IL-10, and TNF-α, though the increases were not as drastic as those seen in the Tg PDFM group (*P*<0.05). The Non-Tg PDFM group did not have a significant increase IFN-γ. G-CSF levels increased non-significantly in all PDFM groups, but decreased in the control group. The control treatment group had significant increases in IFN-γ (*P<*0.001) and TNF-α (*P<0.05*), but not any other cytokine.

**Figure 7:**
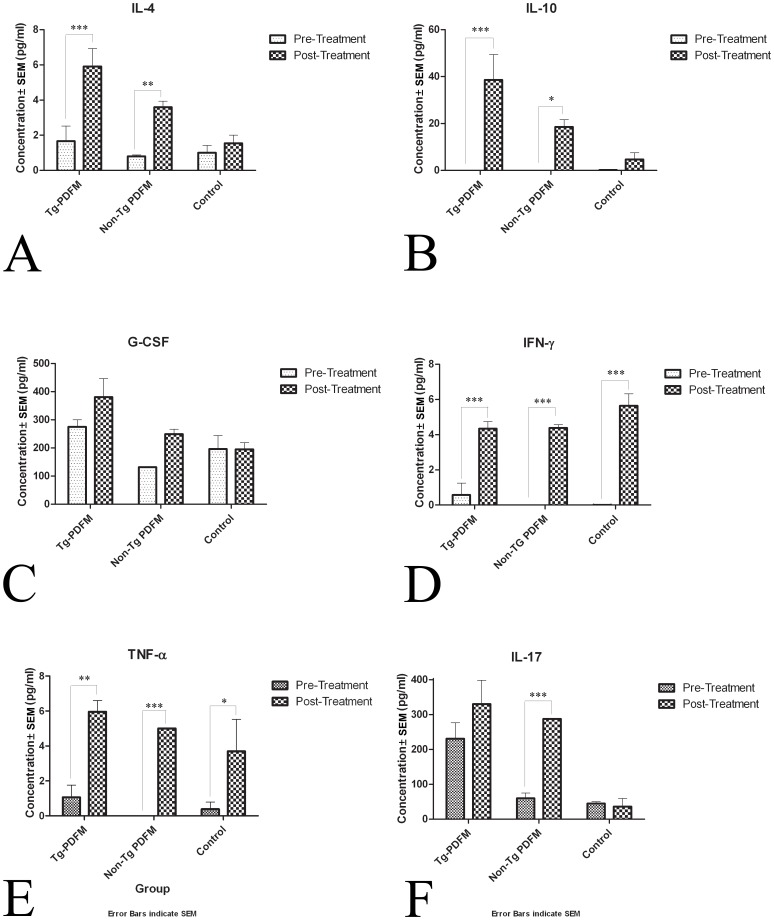
Cytokine expression profile. A) IL-4 increased in Tg PDFM (*P*<0.001) and Non-Tg PDFM (*P*<0.01). B) IL-10 increased in Tg PDFM (*P*<0.001) and Non-Tg PDFM (*P*<0.05). C) GSF increased moderately in the PDFM groups, but not the control. D) IFN-γ increased in all three groups (*P*<0.001) E) TNF-α increased in all three groups (P<0.05). F) IL-17 was elevated in the Non-Tg PDFM group (*P*<0.001).

When comparing post-treatment cytokine levels between groups, the Tg PDFM mice had elevated IL-4, IL-10, IL-17 (*P<*0.001), and G-CSF (*P*<0.01) compared to the control; however, TNF-α and IFN-γ levels were not significantly different among other groups. The Tg PDFM group also had signficantly higher levels of IL-4, IL-10, and G-CSF compared to the Non-Tg PDFM group (*P*<0.05*).* Only IL-17 increased in the Non-Tg PDFM group compared to the control group (*P*<0.001). Cytokine expression results are shown in Figure 7.

As the PDFM group consists of IL-4 cultured DCs, injection of these cells tends to enhance the anti-inflammatory (Th2) response and inhibit the inflammatory (Th1) cascade [37]. Ig Isotyping was done to generate an IgG1/IgG2a ratio. IgG1 production is stimulated by Th2 cytokines, while IgG2a production is stimulated by Th1 cytokines. Total Ig Isotyping analysis showed a drastic increase in IgG1/IgG2a after vaccine administration (*P*<0.05) (Figure, 8A), while the Aβ specific Ig Isotyping did not differ throughout the duration of the treatment.

**Figure 8:**
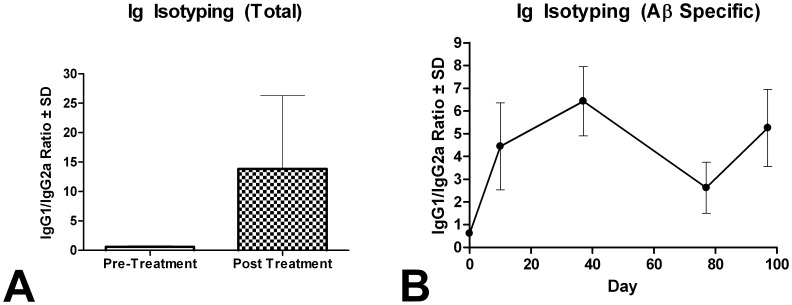
Ig Isotyping. A) Total Ig isotyping. Tg PDFM group showed an increase in IgG1/IgG2a ratio after treatment (*P*<0.05) B) Aβ Specific isotyping. There was no significant difference in Aβ specific isotyping during the duration of the experiment. Note: Ig isotyping was only done for the Tg PDFM group. Aβ specific Ig isotyping was not able to be done at Day 0, as no Aβ antibody was present.

#### PDFM did not cause T-Cell infiltration into the brain

**Figure 9:**
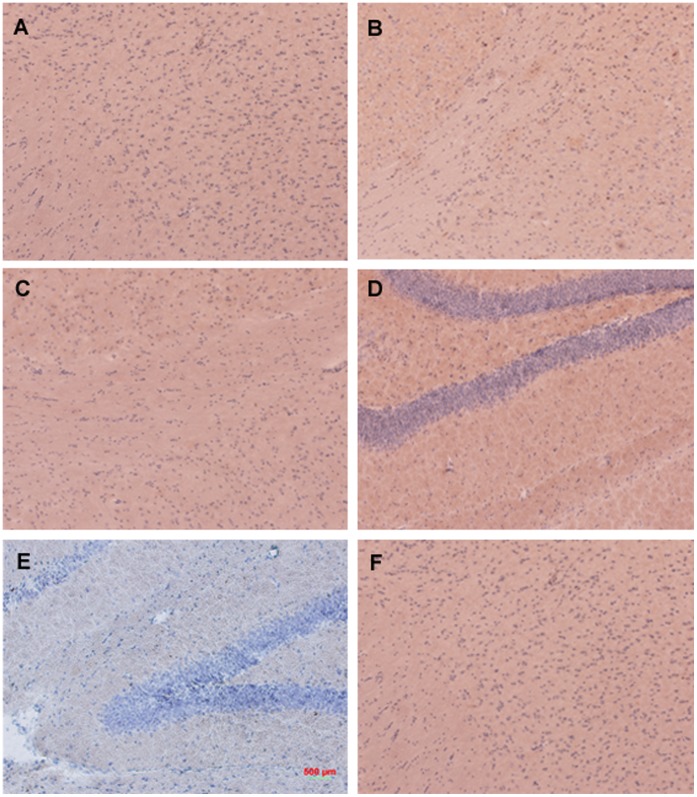
CD3 staining. A) Non-Tg PDFM Cortex B) Tg Control Cortex C) Tg PDFM Cortex D) Non-Tg PDFM Hippocampus E) Tg Control Hippocampus F) Tg PDFM Hippocampus. Some T-Cell infiltration was observed in the hippocampi of the Tg control group, but not in any treatment groups. All images were captured at 10×magnification.

The CD3 staining results clearly show that none of the mice in the Tg PDFM or Non-Tg PDFM group had T-Cell infiltration in the brain. However, mice of the Tg Control group show some T-Cell infiltration (Figure 9).

#### PDFM did not cause vascular hemorrhaging in the brain

**Figure 10:**
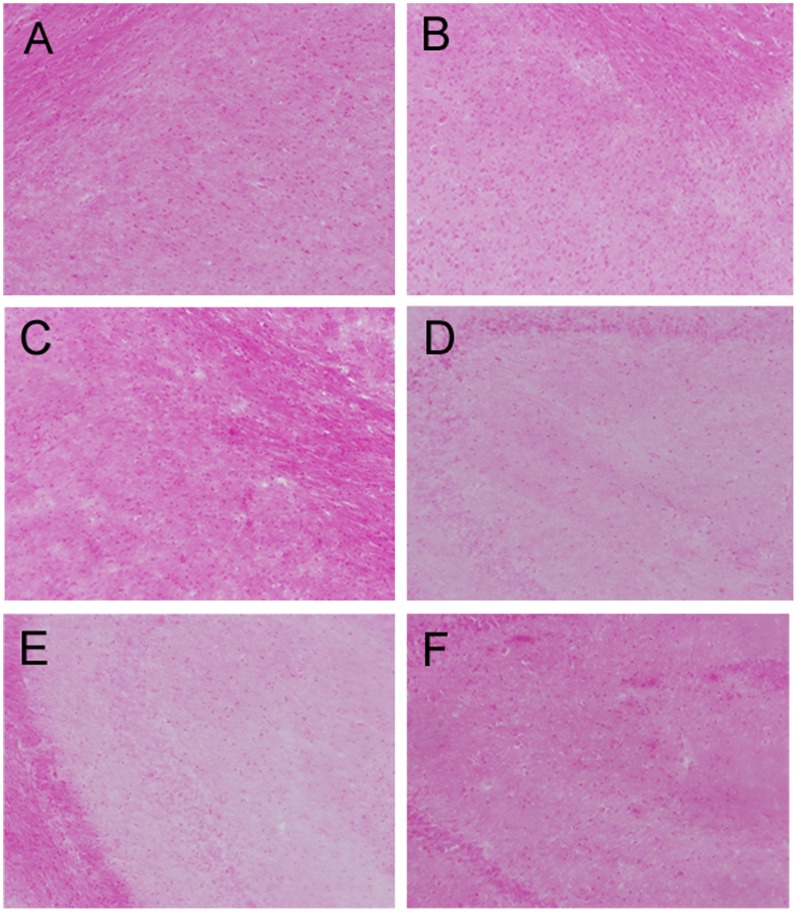
Prussian Blue Staining. A) Non-Tg PDFM Cortex B) Tg control Cortex C) Tg PDFM Cortex D) Non-Tg PDFM Hippocampus E) Tg Control Hippocampus F) Tg PDFM Hippocampus. No vascular bleeding was observed in any mice of any groups. All images were captured at 10×magnification.

Prussian Blue staining was used to look for vascular hemorrhaging in the brain. No hemorrhaging was observed in mice of any group. The results are shown in Figure 10.

## Discussion

Before the advent of 2×Tg AD murine models, single Tg strains expressing mutant amyloid precursor protein (APP) were widely used [38], [39]. Since Aβ peptides are generated directly from the cleavage of APP, increased Aβ levels in these strains eventually result in the formation of amyloid plaques. Our vaccine has shown encouraging results in 1×Tg (PDAPP) mice [30]. However, as drawbacks of the PDAPP model are well documented [40], we decided to study the vaccine in 2×models as well. The 2×Tg model used here (APP+PS1) contains both mutant APP695_sw_ and presenilin-1 (PS1). Introduction of the PS1 mutation into Tg APP mice remarkably increases Aβ_1–42_ levels, accelerating the onset of AD pathology. In APP+PS1 mice, fully formed amyloid plaques are visible by 6 months of age, with plaque formation localized to the cortex and hippocampus [41], [42]. Notably, these mice were the first to show a progressive, age-related decline in cognitive function in the RAWM. At 5–7 months of age, APP+PS1 mice show no deficit in the T4 acquisition trial, but perform worse (though not significantly) in the T5 working memory trail compared to the control (average of 3 errors versus.7 for the control) [34]. At 8 months of age, Ethel reported significant cognitive impairment in the RAWM test [32], and Aβ deposition at this time reaches levels similar to those seen in AD patients (10–12%) [43]. Reports by Puolivali and Arendash et al. show augmented cognitive impairment in the RAWM test at 12 and 15 months respectively [34], [44]. Most importantly, the APP+PS1 model was the first model in which Aβ deposition correlated well with memory loss, a significant finding at the time [45].

Interestingly, these mice show cognitive impairment in the Y-maze at 3 months of age, *prior* to the deposition of amyloid plaques. The cognitive function deficits at this age may be due to the presence of oligomeric Aβ, as the oligomeric (soluble) form of Aβ (as opposed to the insoluble fibrils) have recently been implicated as the neurotoxic agent in AD - implying that AD progression begins prior to plaque formation [46], [47]. In this study, vaccine administration started around 9 months of age, well after the onset of AD pathogenesis (and cognitive decline). This study, therefore, investigates the ability of our vaccine to halt or reverse AD pathology. Prior studies in our lab investigated this vaccine as a preventive treatment for AD in PDAPP mice, with vaccine administration beginning prior to the appearance of cognitive decline and amyloid plaques. The vaccine was able to prevent the progression of AD, but did not give insight to the efficacy of this vaccine when administered after the onset of AD.

The APP+PS1 model was chosen not only due to its accelerated AD pathology, but also because of the inflammatory events observed in the brains of these mice. Since 2005, there has been increased focus on the role of inflammation in AD [48], [49]. Substantial increases in astrocytes and microglia have been observed in APP+PS1 in parallel with increases in Aβ accumulation between 6–18 months of age [50]. As our past studies indicate that DC vaccines provide benefit in part through their immune-modulating ability [30], the neuroinflammatory reactions in APP+PS1 mice provide a viable model to study the interactions between our DC vaccine and the inflammatory events seen in AD patients. Nonetheless, it is important to note that the APP+PS1 model mimics only inflammatory events resulted from Aβ accumulation, therefore there is no immune involvement in AD prior to occurrence of Aβ accumulation.

As past AD vaccines failed in clinical trials due to generation of a Th1 response, we investigated a series of mutated Aβ peptides to determine the *in vitro* immunogenic effect that each had on DCs. Surprisingly, wild-type Aβ itself generated an increased levels of Th1 cytokines in DCs. DC processing of Aβ increased IL-12 and IFN-γ production, while decreasing IL-4– the most typical Th2 cytokine. Many groups have reported DCs or DC-like microglia in the CNS, though the mechanisms of cellular genesis and/or infiltration are still unknown [51], [52]. Our results indicate that Aβ processing by DCs *in vivo* could initiate Th1 activation, triggering the inflammatory events seen in AD patients. A recent study by Ciaramella et al. further corroborates this thought, reporting that DCs isolated from DC patients show a consistent increase in ICAM-1 production – a typical pro-inflammatory molecule. Interestingly, although PWT sensitized DCs are unable to elicit an antibody response when administered as a vaccine, our past studies show that they are still able to reduce Aβ accumulation, implying that sensitized dendritic cells themselves may play a role in reduction of Aβ accumulation through mechanisms separate from antibody mediated clearance [30]. The immune-modulating capabilities of this vaccine provide a strong platform for the study the immune modulating capabilities of the Aβ peptide itself.

Our in vitro study showed that PDFM was optimal for initiating a Th2 response. The PDFM sensitized DC vaccine in this study induces Tg APP+PS1 mice to produce enough antibody titer to signify a notable immune response, even 100 days after inoculation. Although the Tg mice developed a lower antibody titer than the non-Tg cohort, result was expected as Tg mice have been shown to be hyporesponsive to human Aβ [53]. A recent review by Tabira describes that an AD vaccine should have to overcome the problems faced by past efforts. The new vaccine must avoid autoimmune encephalitis, be useful for disease prevention, modify disease course, and provide some sort of cognitive benefit [54]. Our vaccine does all of the above, and has certain advantages over other AD vaccines in development. Firstly, both active and passive vaccines for AD are in development. Although both have shown efficacy in plaque removal, active immunization requires far fewer treatments and provides a longer lasting antibody response at a lower cost. Although active immunization does have drawbacks (ex. potential side effects: fever, swelling, etc.), the *ex vivo* antigen presentation in DC vaccine likely ameliorates these problems. This *ex vivo* antigen presentation also circumvents the need for direct Aβ injection, which could cross the blood brain barrier but act as a “seed” for enhanced Aβ fibril formation [55]. Our vaccine avoids further aggression of Aβ oligomerization via introduction of this Aβ “seed.” Additionally, cell therapies often face the problem of graft versus host disease. As DCs can be donated and cultured from the patient themselves, adverse effects are not likely [56]. Finally, if prepared correctly, our DC vaccine has the distinct advantage of being able to produce antibodies specific to oligomeric Aβ, further mitigating the possibility of adverse effects. Currently, the physiological function of (monomeric) Aβ has not been identified. However, genetic analysis has shown it is highly conserved among mammals, indicating it may have an important physiological role. If Aβ is allowed to oligomerize prior to the *ex vivo* antigen presentation, the dendritic cell would present oligomeric Aβ to antibody producing cells, producing more specific antibodies than capable by contemporary active vaccines.

The AN-1792 clinical trial exhibited the variability of subjects’ antibody responses to active Aβ vaccines – some patients had robust, lasting responses, while others showed no antibody response at all [15]. Moreover, crossbred mice, such as the transgenic mice used in this experiment, can have significant variability between individuals. As such, the authors believe quantification of antibody responses and stratification of individual mice as normal responders or low responders is necessary to provide a complete picture of vaccine efficacy. The 75^th^ percentile was used as the indicative value for strength of antibody titer. The 75^th^ percentile value is a more indicative value than the mean because it is not subject to skewing from one high titer value. Moreover, only mice with sustained antibody titers will have high 75^th^ percentile values, since multiple elevated titers are needed to keep the 75^th^ percentile value up. As the antibody mediated effect is one that provides protection over a long period of time, mice with high 75^th^ percentile values are more likely to gain protective value from the vaccine and thus can be classified as “high responders.” The behavioral studies show that the PDFM vaccine is able to rescue cognitive function in APP+PS1 mice to levels similar to Non-Tg mice. However, the cognitive benefit ascertained from treatment was not statistically significant compared to the Tg Control group. Administration of the vaccine began at 9 months of age, well after the initiation of AD pathogenesis. Taking into account reports of the neurotoxic effects of oligomeric Aβ, it is reasonable to believe that the neuronal damage and cognitive loss began prior to the beginning of treatment. The cognitive benefit seen in our study is likely due to the protective effect of our vaccine in inhibiting further disease progression, as opposed to reversal of initiated cognitive decline. Our findings are consistent with the suggestion that immunotherapy against AD may be more powerful as a preventive treatment as opposed to a therapeutic treatment [57]. In earlier studies, we investigated this vaccine as a preventive measure against AD in PDAPP mice. PDAPP express many of the neuropathological features of AD as early as 6 months of age [58], at which point they show significant impairment in the Morris Water Maze (MWM), open field, radial arm maze, operant bar pressing, and visual object recognition testing compared to age matched controls.

In PDAPP, the extracellular Aβ deposition and insoluble extracellular plaque formation occurs shortly after 9 months of age. Similar to the APP+PS1 model cognitive function deficits appear before the appearance of fully formed amyloid plaques [59]. When treatments in PDAPP were started at 4 months of age, *prior to* the appearance of cognitive decline and amyloid plaques, the treatment group performed significantly better than the Tg Control group three months later. Treatment at an earlier age cleared and inhibited formation of oligomeric Aβ, explaining the significant gain in cognitive function. The lack of statistically significant in this experiment is not due to the efficacy of the treatment itself, but more likely to the period of administration.

Interestingly, the low responder group performed significantly worse than the Tg Control with regards to both error and latency. Though we do concede the sample size is small, the data does suggest that progression of AD hastened in low responders. Mice that were not able to develop a strong antibody titer in response to the vaccine likely have worsened immune function than mice that had a strong response. Though the connection between the immune system and AD pathogenesis is not clear, it has been suggested that the immune system plays a protective role against AD pathogenesis, or even that lack of immune function could be a causative factor of AD progression [60]. The worsening of AD progression in low responders may be due to the lack of immune function signified by the low antibody response. The correlation between immune function and rate of cognitive decline in AD patients’ clearly needs to be further explored.

**Figure 11:**
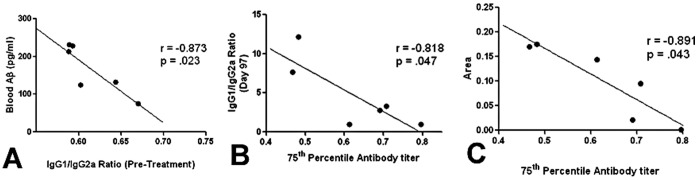
Correlationary analysis. A) Antibody titer is inversely correlated with Aβ burden (*P*<0.05) B) Pre-treatment IgG1/IgG2a ratio is inversely correlated with pre-treatment Blood Aβ (*P*<0.05) C) IgG1/IgG2a ratio is positively correlated with antibody titer (*P = *0.19) Stars indicate statistical significance: * *P*<0.05, ** *P*<0.01, *** *P*<0.001.

The results show that DC vaccine use resulted in a significant reduction in Aβ burden, as mice of the treatment group had 57% and 65% decreases in the cortex and hippocampus respectively compared to the control upon histological analysis. It is important to note that Congo red staining does not measure total Aβ levels, but rather measures insoluble amyloid. However, previous studies have shown that the total Aβ does not always correlate with vaccine efficacy [10]. Moreover, there is a strong correlation between antibody titers and amyloid burden; mice with higher antibody titers generally have a lower Aβ burden (Figure 11A, r = −0.891, p<0.05). The most obvious mechanism of DC vaccine efficacy is through the antibody production. Thus far, four potential pathways have been hypothesized to play a role in antibody-mediated clearance of Aβ. These mechanisms were laid out in a recent review by Morgan [61]. The first mechanism suggests antibodies opsonize targets, resulting in macrophage related plaque clearance [13]. Past studies on this DC vaccine have shown CD45 (microglial) activation, perhaps providing credence to this hypothesis. However, only a small fraction of circulating antibodies cross the blood brain barrier, leading to the assumption that binding of antibodies to Aβ in the blood causes efflux of Aβ from the brain, a so called “peripheral sink” [12]. The third mechanism suggests a conformational change that prevents oligomerization as a result of Aβ peptide and antibody interactions [62]. The final mechanism theorizes that Fc receptor (FcRn) mediated efflux of Ab-Ag complexes across blood brain barrier augment Aβ clearance [63]. It is important to note that these mechanisms are not mutually exclusive. It is possible (and probable) that antibodies mediate clearance by activating multiple pathways. Viewing our results through the scope of these mechanisms, even the relatively low antibody titers seen in the PDFM group at Day 100 should have an appreciable effect on Aβ clearance. Studies have shown that a 1∶1000 antibody to Aβ peptide ratio still results in clearance [64]. Although an antibody titer was present throughout the duration of the experiment, it is likely there was significant variability between booster injections. Figure 2b shows the antibody titer 24 days after the initial inoculation - antibody titer has significantly decreased since day 10. The variability in antibody levels between booster injections should not affect the efficacy of antibody mediated clearance due to the different mechanisms through which the antibodies work. Moreover, modulation of blood Aβ levels by vaccine use may be due to antibody binding and clearance of Aβ in the blood, perhaps initiating formation of a peripheral sink.

In characterizing the safety of our vaccine, we looked for generation of a Th2 response, the absence of microhemorrhaging, and the absence of T-cell infiltration. The immune response was evaluated through the cytokine expression profile and Ig Isotyping. The Tg mice had a more robust change in cytokine expression than the Non-Tg cohort, a phenomenon that may be due to the dynamic interplay between AD pathology and the immune system. Mice of the Tg PDFM group had an upregulated anti-inflammatory Th2 response, with an inhibited pro-inflammatory Th1 response. IFN-γ and TNF-α, the most typical Th1 cytokines in sera, were not increased compared to the control group. The cytokine profile of the DC vaccine groups showed no increase in Th1 profile. The obvious implication, that the adjuvant, but not the production of anti-Aβ antibodies, induced Th1 response in AN-1792 is an encouraging indicator that this vaccine may overcome the problems of past anti-Aβ vaccines. The PDFM group also showed an increase in IL-4 and IL-10 (typical Th2 cytokines) compared to the control. The IgG1/IgG2a ratio also increased significantly after treatment, confirming production of a Th2 response. Presumably, treatment of the DC’s with IL-4 in culture prior to vaccine administration augmented the release of Th2 cytokines by the DCs, initiating a Th2 cascade. In past studies, we compared the cytokine expression profiles between mice given a DC vaccine injection and those given Aβ+adjuvant [30]. The mice injected with the adjuvant had significantly higher TNF-α and IFN-γ levels in their blood than the control, while DC vaccine mice didn’t. The fact that the APP+PS1 PDFM mice do not have high levels of those cytokines bolsters our belief that this vaccine can avoid the problems seen in AN-1792. Furthermore, no T-cell infiltration or brain microhemorrhaging was observed upon histological analysis. As the vaccine did not initiate global inflammation, cause microhemorrhaging, or initiate T-cell infiltration into the brain, it appears safe. Furthermore, additional cognitive benefit may be derived from the vaccines ability to push a primarily Th2 response, as introduction of Th2 T-cells have been shown to improve cognitive function [65].

Additionally, the concentration of sera IL-17 in the PDFM group elevated significantly in this study and sustained until the end of the experiment. Elevation of IL-17 levels is essential for breakthrough in immune tolerance or implied generation of autoimmune response. The role of IL-17 in production of anti- Aβ antibodies needs to be characterized further. It is our belief that DC’s increase Th2 (anti-inflammatory) response, further opposing the immune response that caused adverse conditions in the AN-1792 trial.

One other important observation in the cytokine profile of the PDFM that may provide mechanistic insight into the effect of DC Vaccines was the concentration levels of G-CSF. G-CSF can mobilize activation of neutrophilic granulocytes and stimulate proliferation and differentiation of hematopoietic cells. Samchez-ramos *et al.* [66] have reported G-CSF can be used as therapy for memory impairment in AD-Tg mice. The mechanism is believed to be mobilization of hematopoietic stem cells followed by implantation in the brain, facilitating neural regeneration. Elevation of G-CSF concentration in the PDFM group is may enhance therapeutic effects.

Statistical analysis of the data brought out two other important correlations. Firstly, pre-treatment IgG1/IgG2a ratio is inversely correlated with blood Aβ levels (Figure 11B, r = −0.873, *P = *0.023.) In other words, mice which initially have T-cell repertoires skewed towards Th1 T-cells, are likely to have lower blood (and presumably total) Aβ levels. It has been suggested that rheumatoid arthritis patients, those with chronic (Th1) inflammation, have a significantly lower risk of developing Alzheimer’s disease [67], [68]. Our results are consistent with the finding that an inclination towards a Th1 T-cell reaction may lower Aβ levels. Secondly, total IgG1/IgG2a ratio post treatment was positively correlated with antibody titer (Figure 11C, r = 0.617, *P = 0*.19). Mice with a more Th1 skewed T-cell response had more robust antibody titers. Though the effects of the immune system on Alzheimer’s disease are not clear, it is reported that administration of Th2 T-cell can provide cognitive benefit to Alzheimer’s mice. This gives credence to the hypothesis that immune balance may play a role in Alzheimer’s progression.

### Conclusions

Our DC cell therapy was capable of producing a significant antibody titer, initiating amyloid plaque clearance, and providing cognitive benefit to APP+PS1 mice. The cell therapy also proved to be safe, as it did not initiated an inflammatory Th1 response, and did not cause microhemorrhaging or T-cell infiltration into the brain. This antigen sensitized DC cell therapy brings together many of the traits needed in optimization of immunotherapy for AD, and in the future may be a viable option for the treatment of AD.
