# Correction: Cell Therapy: A Safe and Efficacious Therapeutic Treatment for Alzheimer’s Disease in APP+PS1 Mice

**DOI:** 10.1371/journal.pone.0303619

**Published:** 2024-05-09

**Authors:** Neel R. Nabar, Fang Yuan, Xiaoyang Lin, Li Wang, Ge Bai, Jonathan Mayl, Yaqiong Li, Shu-Feng Zhou, Jinhuan Wang, Jianfeng Cai, Chuanhai Cao

In Fig 9 of [1], panel A was erroneously duplicated in panel F. In the corrected version of Fig 9, provided here, the correct image from the original experiment is shown in panel F. The underlying image data for Fig 9 are in S1 File. The authors additionally provide an annotated version of Fig 9E to assist visualization of T-Cell infiltration in this panel (S2 File).

The underlying data supporting other results in the article are no longer available due to deletion in accordance with institutional policy.

Reference 35 in the article [1] is incomplete. The correct reference is:

35. Postupna N, Rose SE, Bird TD, Gonzalez-Cuyar LF, Sonnen JA, et al. (2012) Novel Antibody Capture Assay for Paraffin-Embedded Tissue Detects Wide-Ranging Amyloid Beta and Paired Helical Filament-Tau Accumulation in Cognitively Normal Older Adults. Brain Pathol. 22(4): 472–484. doi: 10.1111/j.1750-3639.2011.00542.x

The authors apologize for the errors in the published article.

**Fig 9:**
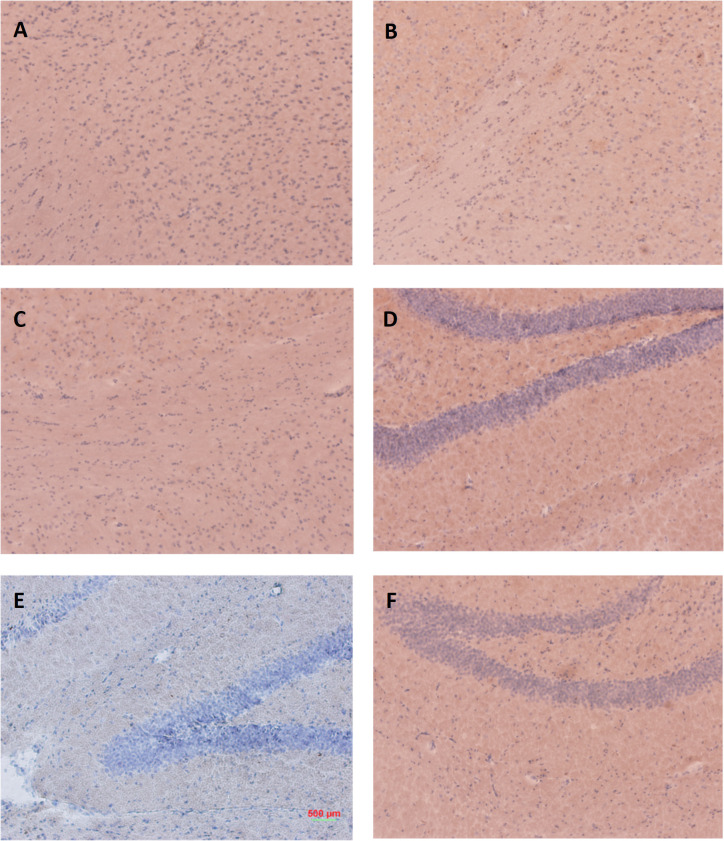
CD3 staining. A) Non-Tg PDFM Cortex B) Tg Control Cortex C) Tg PDFM Cortex D) Non-Tg PDFM Hippocampus E) Tg Control Hippocampus F) Tg PDFM Hippocampus. Some T-Cell infiltration was observed in the hippocampi of the Tg control group, but not in any treatment groups. All images were captured at 10×magnification.

## Supporting information

S1 File Underlying images for Fig 9. (ZIP)

S2 File Annotated version of Fig 9E. (JPG)
