# Supplementary figures and images for: Correction: Cell Therapy: A Safe and Efficacious Therapeutic Treatment for Alzheimer’s Disease in APP+PS1 Mice
Source: PLoS One. 2024 May 9;19(5):e0303619. doi: 10.1371/journal.pone.0303619 (PMC11081276; doi:10.1371/journal.pone.0303619)

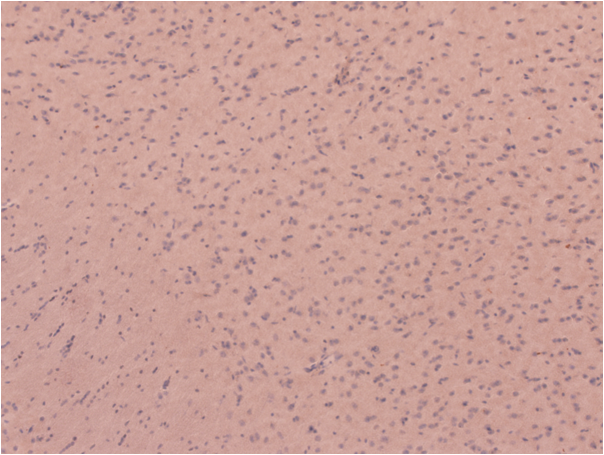

Supplement: S1 File — (ZIP) [file pone.0303619.s001.zip › S1 File/Fig9A Non Tg CX.tif]

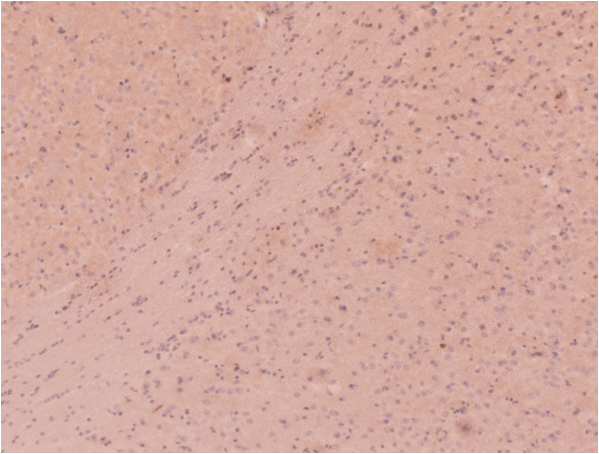

Supplement: S1 File — (ZIP) [file pone.0303619.s001.zip › S1 File/Fig9B Tg non Treat CX.tif]

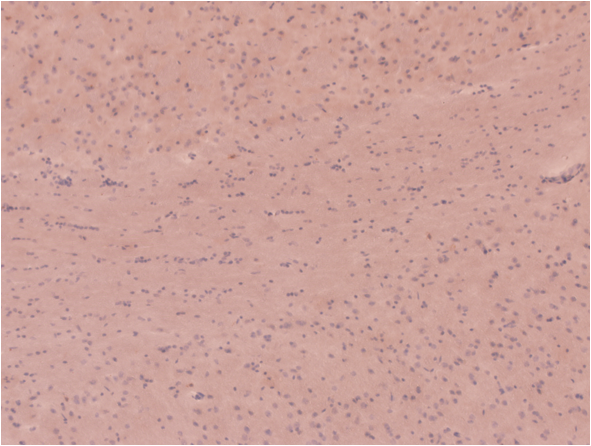

Supplement: S1 File — (ZIP) [file pone.0303619.s001.zip › S1 File/Fig9C Tg Treat CX.tif]

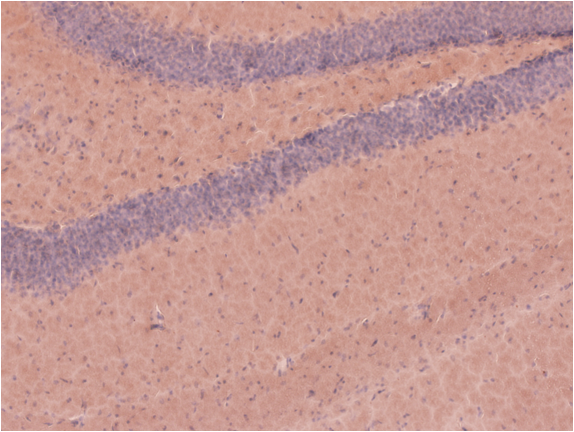

Supplement: S1 File — (ZIP) [file pone.0303619.s001.zip › S1 File/Fig9D Non Tg hip.tif]

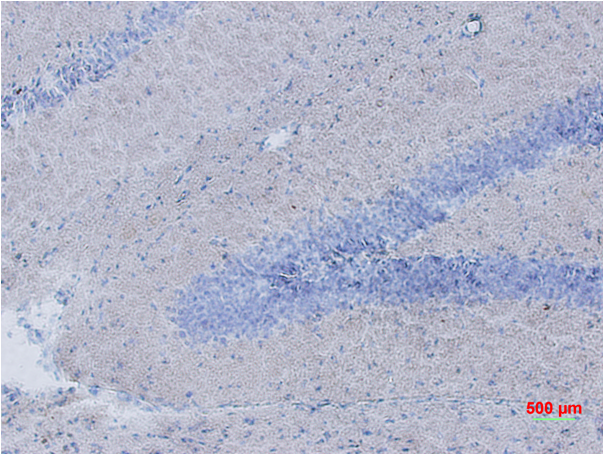

Supplement: S1 File — (ZIP) [file pone.0303619.s001.zip › S1 File/Fig9E Tg non treat hip.tif]

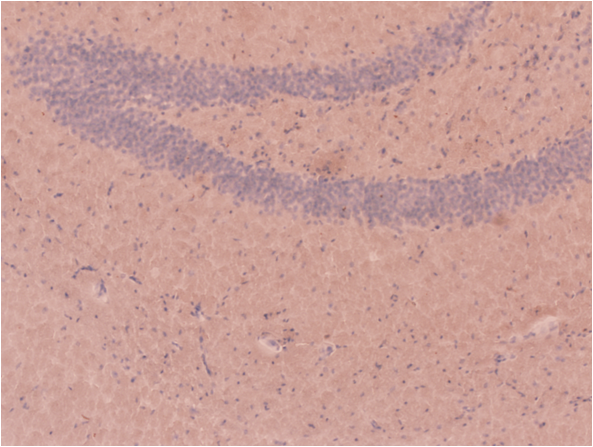

Supplement: S1 File — (ZIP) [file pone.0303619.s001.zip › S1 File/Fig9F Tg treat hip.tif]

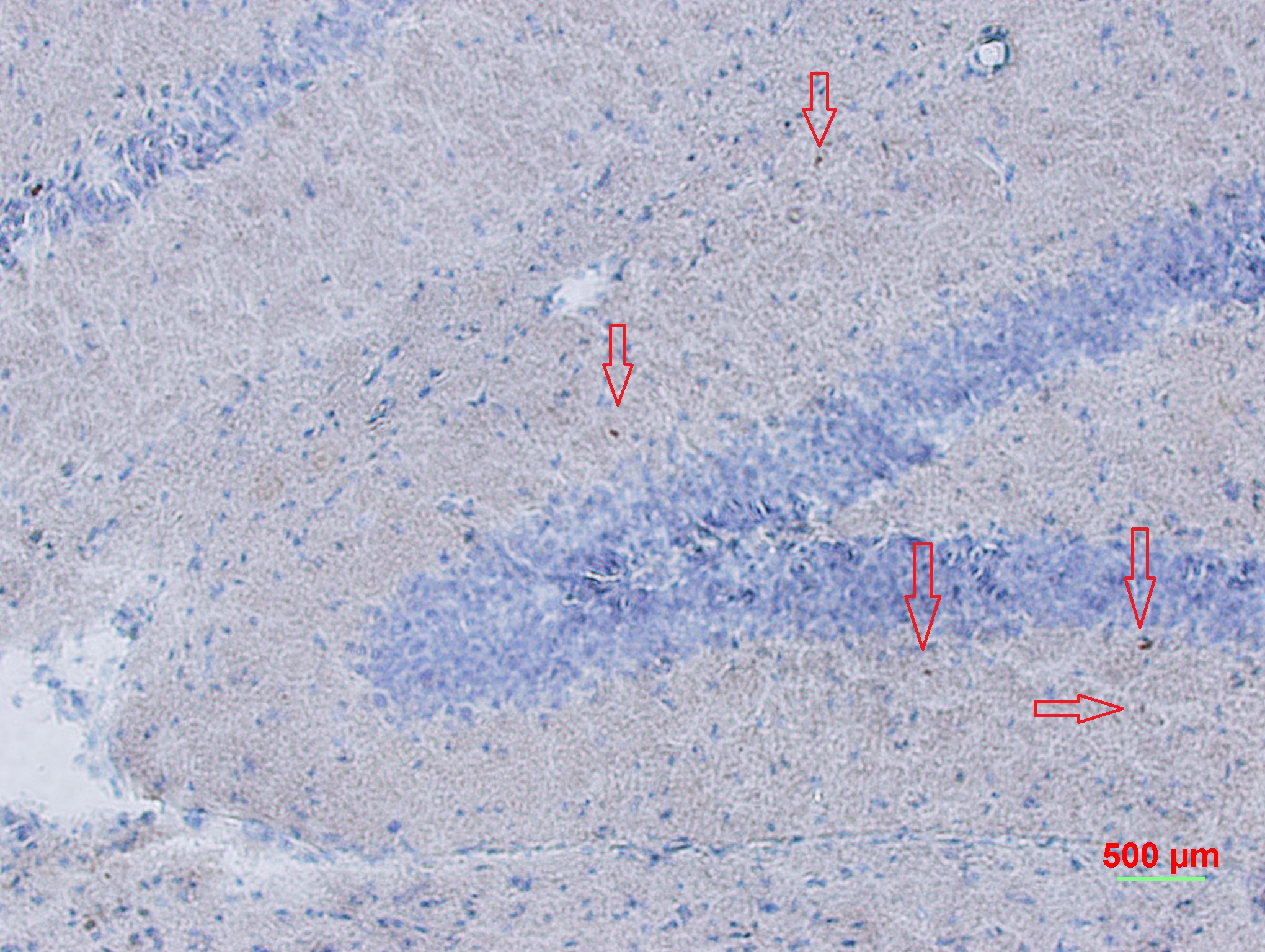

Supplement: S2 File — (JPG) [file pone.0303619.s002.jpg]
